# Mitchell-Riley Syndrome Due to a Novel Mutation in RFX6

**DOI:** 10.3389/fped.2019.00243

**Published:** 2019-06-18

**Authors:** Mohammed Abdulmageed Kambal, Doha Ayed Al-Harbi, Areej Rashed Al-Sunaid, Mohsen Suliaman Al-Atawi

**Affiliations:** ^1^Division of Pediatric Gastroenterology, Pediatric Department, King Khalid University Hospital, King Saud University, Riyadh, Saudi Arabia; ^2^Division of Pediatric Endocrinology & Diabetes, King Abdullah Specialist Children's Hospital, Riyadh, Saudi Arabia; ^3^Division of Gastroenterology, Hepatology, and Nutrition, King Abdullah Specialist Children's Hospital, Riyadh, Saudi Arabia; ^4^King Abdullah Specialist Children's Hospital, King Saud bin Abdulaziz University for Health Sciences (KSAU-HS), Ministry of National Guard Health Affairs, Riyadh, Saudi Arabia

**Keywords:** duodenal atresia, congenital, neonatal diabetes mellitus (NDM), cholestasis, regulatory factor X6 (RFX6), c.983A>T p.(asp328Val), homozygous, MODY (maturity-onset of diabetes in the young)

## Abstract

We report a Saudi girl who presented at birth with neonatal diabetes, duodenal atresia, and progressive cholestasis. After other gene testing was negative, the clinical diagnosis of Mitchell-Riley syndrome was ultimately considered and further genetic analysis revealed a novel missense homozygous variant in RFX6: c.983A>T (p.asp328Val). Despite intensive management, the patient died from severe *Klebsiella pneumoniae* sepsis at 5 months of age. This rare syndrome should be suspected in any neonate with hyperglycemia complicated by intestinal atresia and/or progressive cholestasis that could suggest biliary hypoplasia. Early recognition and diagnosis through genetic testing are essential for guiding aggressive clinical management as well as family counseling, particularly in light of the high possibility of early death in this highly complex disorder.

## Case Report

The patient was a twin A dizygotic girl who was the product of an *in-vitro*-fertilization (IVF) pregnancy in a consanguineous couple from Saudi Arabia. The father is a 33 years old Saudi male and the mother is 28 years old. The mother has normal glucose profile during her routine antenatal care and the father is not diabetic. The antenatal ultrasound showed a dilated bowel suggesting the possibility of duodenal atresia. The patient was born prematurely at 30 weeks of gestation by an emergency Cesarean section. Her Apgar score was 6, and 8 at the first and fifth minute, respectively. Her birth growth parameters were as follows weighed was 1.1-kg, head circumference 26 cm, length was 38 cm. No dysmorphic features were observed. At birth, the suspected duodenal atresia was confirmed, and she underwent a surgical repair, during which a jejunal cyst was found and removed.

During her third day of life, she developed severe and persistent hyperglycemia ranging from 16 to 26 mmol/L, which did not improve even after substantial reduction on glucose concentrations in her total parenteral nutrition (TPN) along with very low insulin level which was <2 μIU/mL (laboratory reference: 3.2–16.3 μIU/ml) and C-peptide level <0.1 ng/mL (Laboratory reference: 0.8–4.2 ng/mL). The diagnosis of neonatal diabetes was made, and she was commenced on a continuous intravenous insulin infusion because the amount of her subcutaneous fat was not adequate for subcutaneous insulin administration. Despite meticulous insulin dosage adjustment her blood sugar was always high and was in the range from 12 to 14 mmol/L.

The cause of her neonatal diabetes was investigated thoroughly. The autoantibodies against pancreatic islets cells, insulin, and glutamic acid decarboxylase (GAD) were negative. Genetic testing for the common gene mutations causing neonatal diabetes namely ABCC8 and KCNJ11 were negative.

At the third week of her neonatal course the patient devolved a progressive cholestasis and her stool reported to be white in color. Her initial and subsequent liver function tests were consistent with extrahepatic cholestasis and are summarized in Table 1.The patient abdominal ultrasound revealed a normal homogenous liver with small, contracted gallbladder, and tiny cyst at porta hepaticus. HIDA scan showed no contrast trace in the bowel after 24 h. Due to the neonate's unstable condition, no further investigations were done regarding the hepatobiliary system. When she started feeding orally, she developed diarrhea, so TPN was introduced.

**Table 1:** Liver function tests.

**Test**	**Reference value**	**At birth**	**1st week**	**2nd week**	**3rd week**
Bilirubin -Total	<34 μmol/L	35.2	47.8	51.3	69.8
Bilirubin -Direct	<8.6 μmol/L	11	24.6	43.7	61.5
ALP	134–518 U/L	97	162	229	353
AST	5–34 U/L	14	17	29	57
ALT	5–55 U/L	8	13	26	46
GPT	9–36 U/L	128	427	694	725

*ALP, Alkaline phosphatase; AST, Aspartate aminotransferase; ALT, Alanine aminotransferase; GPT, Gamma-glutamyl transferase*.

After considering her clinical course the diagnosis of Mitchell-Riley syndrome (MRS) was entertained and was confirmed by genetic testing for the regulatory factor X6 (RFX6) gene which revealed a homozygous mutation c.983A>T p. (Asp32Val), confirming the diagnosis.

At 5 months, the patient died from severe *Klebsiella pneumoniae* sepsis.

## Discussion

Mitchell-Riley syndrome (MOM # 615710) is a syndrome characterized by neonatal diabetes, pancreatic hypoplasia, intestinal atresia, and gallbladder hypoplasia or aplasia, chronic diarrhea, intrauterine growth restriction, and consanguinity.

Mitchell et al. reported five pediatric patients with neonatal diabetes mellitus (NDM) resulting from pancreatic hypoplasia, who also presented with intestinal atresias and hypoplastic gallbladder. All the reported patients had low birth weight, but none had had dysmorphic features (1–4).

This rare syndrome is caused by a mutation involving the RFX6 gene, which has an important biological role in the development of intestine, gallbladder as well insulin-producing pancreatic beta-cells (5).

Several mutations have been identified in the RFX6 gene, and they are summarized in Table 2. In our case, a RFX6 gene analysis identified a homozygous variant c.983A>T p.(asp328Val), which was a previously unreported mutation.

**Table 2:** The reported RFX6 gene mutations.

**RFX6 gene mutation**	**Gastrointestinal defect**	**Pancreatic abnormality**	**Onset of diabetes (age)**	**Gall bladder anomaly**	**References**
C.380+2T4C homozygous (in 2 cases)	Duodenal and jejunal atresia	Annular	1–2 days	hypoplasia	Mitchell et al. (1)
C.672+2T4G/c.224-12A4G Compound heterozygous	Duodenal web with malrotation	Small	2 days	hypoplasia	Mitchell et al. (1)
c.649T4C homozygous	Duodenal atresia with malrotation	Not reported	8 days	agenesis	Chappel et al. (2)
c.542G4A homozygous	Duodenal and jejunal atresia with malrotation	Hypoplasia	14 days	agenesis	Martinovici et al. (3)
c.781-2_787delAGGTT-GATAinsG homozygous	Duodenal and jejunal atresia with malrotation	Annular	1 day	agenesis	Smith et al. (4)
p.K260T (also disrupts intron 7 splice donor site) Homozygous	Duodenal atresia with malrotation	Annular	1 day	agenesis	Spiegel et al. (5)
c.541C>T, p.R181W Homozygous	Duodenal atresia	Annular	2 days	agenesis	Concepcion et al. (6)
c.1153C>T p.Arg385^*^ Homozygous	Duodenal atresia	Hypoplasia	2 days	agenesis	Zegre et al. (7)
c.983A>T p.(Asp328Val) Homozygous	Duodenal atresia	Normal^*^	3 days	hypoplasia	This case

* *No gross anomaly*.

Progressive cholestasis due to gallbladder hypoplasia or aplasia is an essential clinical feature of this syndrome, and its presentation in the contest of a neonatal diabetes should be a diagnostic clue for the treatment team. Indeed, it was the constellation of the triad of intestinal atresia, neonatal diabetes, and cholestasis that led us to the correct diagnosis.

Almost all features described in this syndrome were present in our case. Typically, the duodenal atresia is the most common site and reported in all reported patients, but the lesion could involve any part of the gastrointestinal tract (6, 7). In our case, this anomaly was suggested by prenatal ultrasound and was confirmed and corrected surgically postnatally.

The pancreatic abnormalities in MRS is heterogenous in nature it cloud be seen as an isolated anatomical anomaly like annular or small-sized pancreas or could be limited to the pancreatic endocrine function or a combined endocrine-exocrine deficiencies.This diversity in the pancreatic involvement in MRS probably reflecting the type of mutation involving RFX6.Ceratain mutations resulting in a combined endocrine-exocrine pancreatic deficiency and other saving the pancreatic exocrine function despite severe endocrine deficiency which is typically revealing as non-immune diabetes with its onset ranging from neonatal diabetes to MODY with no evidence of exocrine deficiency. When MRS has its onset in the neonatal or infancy period combined endocrine-exocrine deficiencies are typically evident. Our patient has no gross anatomical anomaly involving her pancreas as seen during the surgical repaired for her intestinal anomaly; however, she has the clinical evidence of severe combined pancreatic deficiencies.

The diabetes in this syndrome is due to pancreatic hypoplasia or beta-cell deficiency and usually onsets in the first few days after birth. In our case, the diagnosis of neonatal diabetes was made on the third day of her life. But the onset of diabetes could be delayed until early childhood due to residual activity of the RFX6 gene (7–11). Heterozygous RFX6 mutations appear to cause a certain form of Maturity-onset diabetes of the young (MODY) (12–15).

The exocrine deficiency was evident upon a trail of oral feeding at second week of her life where she developed severe diarrhea. Her stool described to be white throughout her course. The patient failure to gain weight was evident despite maximizing her caloric intake by adequality prepared TPN, she did not gain any weight, her average weight gain per week was in the average of 30–50 g/week and was not evident in every week and her weight chart indicate several weeks with negligible weight gain by the time of her death at age of 5 months her weight was only 1,500 g. We believe that her severe failure to thrive is rather complex and likely due to both severe combined pancreatic deficiencies in addition to her serve cholestasis.

Mitchell-Riley syndrome should be in the differential diagnosis of any neonate presenting with neonatal diabetes followed shortly by biliary-like progressive cholestasis and RFX6 gene should be immediately tested to confirm the diagnosis and this would help to avoid unnecessary steps in the clinical management like requesting additional genetic testing or performing a liver biopsy. Early diagnosis is crucial for early family counseling about unfavorable outcome.

Cases reported with neonatal onset of this syndrome often have profound gastrointestinal and hepatobiliary defects that in addition to severe insulin-deficient diabetes make them very difficult to manage successfully and they have a high risk of death.

## Ethics Statement

Informed consent was signed for publication.

## Author Contributions

MK, DA-H, AA-S, and MA-A: study design. MA-A: data collection and drafting.

### Conflict of Interest Statement

The authors declare that the research was conducted in the absence of any commercial or financial relationships that could be construed as a potential conflict of interest.
